# A massive experiment on choice blindness in political decisions: Confidence, confabulation, and unconscious detection of self-deception

**DOI:** 10.1371/journal.pone.0171108

**Published:** 2017-02-14

**Authors:** Andrés Rieznik, Lorena Moscovich, Alan Frieiro, Julieta Figini, Rodrigo Catalano, Juan Manuel Garrido, Facundo Álvarez Heduan, Mariano Sigman, Pablo A. Gonzalez

**Affiliations:** 1 Laboratorio de Neurociencia, Universidad Torcuato Di Tella, Buenos Aires, Argentina; 2 Consejo Nacional de Investigaciones Científicas y Técnicas (CONICET), Buenos Aires, Argentina; 3 El Gato y La Caja, Buenos Aires, Argentina; 4 Universidad de San Andrés, Buenos Aires, Argentina; 5 Universidad de Vigo, Vigo, España; University of Vermont, UNITED STATES

## Abstract

We implemented a Choice Blindness Paradigm containing political statements in Argentina to reveal the existence of categorical ranges of introspective reports, identified by confidence and agreement levels, separating easy from very hard to manipulate decisions. CBP was implemented in both live and web-based forms. Importantly, and contrary to what was observed in Sweden, we did not observe changes in voting intentions. Also, confidence levels in the manipulated replies where significantly lower than in non-manipulated cases even in undetected manipulations. We name this phenomenon unconscious detection of self-deception. Results also show that females are more difficult to manipulate than men.

## 1. Introduction

How accurate are the explanations we offer ourselves for the things we do and the choices we make? As Moore and Haggard pointed out [1], this was a question famously tackled by Nisbett and Wilson (1977) in their seminal article “Telling more than we can know: Verbal reports on mental processes” [2].

Stage magicians have exploited for centuries the inability to recognize the external factors that drive our choices. The word “forcing” is used in magic to describe the act in which the performer forces an option to the participant, who nonetheless reports to have chosen freely among all possible options. As with other central tenets of the cognitive underpinnings of magic [3, 4], in psychological forcing the performer profits from the fact that only little and often distorted information is available to the spectator’s introspective constructs. We recently proposed a method to quantitatively measure the capacity of a subject to identify which features of stimuli play a significant role in conditioning her choices [5]. The aim of the current study is to determine a subject’s ability to recognize the elements of internal argumentation that lead to choices.

Research originated in split brain patients and then extrapolated to normal subjects suggests that we are often blind to the thoughts, emotions and deliberations that lead to a decision [6]. In the Choice Blindness Paradigm (CBP), participants fail to notice mismatches between their intended choice and the outcome they are presented with, while nevertheless offering reasons to justify a choice they never made [7–12]. In the CBP seminal article [7], participants chose which of two female faces they considered more attractive. The ‘chosen’ photograph was then presented a second time to the participant, who had to offer a justification for choosing that photograph. Unbeknownst to the participant, the experimenters intermittently swapped the photograph that was chosen for the un-chosen one. Interestingly, Johansson et al. found that, when they presented to the participants a photograph they had not chosen, they would nevertheless offer a justification for that ‘choice’. Choice Blindness is also observed in the domain of moral and political choices [13, 14]; people often use their answers in a manipulated survey as anchors for their justifications. Respondents confabulate reasons for manipulated answers that they did not actually gave, showing a marked introspective blindness to the internal processes leading to a moral or political judgment. This demonstrates that self-deception and self-confabulation are a common feature in how we make decisions and justify them afterwards [15].

In this article, we combine the CBP with confidence reports to assess the extension and scope of self-confabulations on the internal factors leading to a decision. We rely on a long tradition of psychological studies on the performance-confidence relationship to quantify introspective accuracy [16, 17] and we measure the effect of individual variability in introspective accuracy on the internal factors influencing our choices on political or moral issues. In addition, we measure the difference in confidence levels expressed in manipulated (M) and non-manipulated answers (NM) as a proxy to explore whether self-deception is unconsciously detected. We expect instances in which participants will not ask to restore the manipulated answer, since they are not aware of the manipulation, but in which responses might still reflect a change in confidence in the modified answers compared to the non-manipulated ones. We refer to this as an unconscious detection, which only entails that the participants’ actions indicate lack of awareness of the manipulation.

We conducted two experiments: one using a web-based portable platform and one with face-to-face questionnaires in more traditional conditions comparable to previous CBP experiments. The first experiment had the advantages of enabling fast data gathering through desktops, tablets and mobile phones, and of allowing for a dramatic increase in the number of participants compared with previous works on this area. In the second experiment, pedestrians were engaged in a conversation by three trained magicians who acted as researchers on political decisions in downtown Buenos Aires one week before the last presidential election, allowing us to compare and contrast web-based and face to face results.

### 2. Swedish and Argentine CBP experiments in comparative perspective

Based in the model of an experiment done in Sweden in 2010 [14], we adapted the questionnaire and conducted two different experiments before the 2015 presidential election in Argentina. The experiment from Lund asked a number of questions regarding vote intention, political engagement, and other related issues. Respondents were also asked to locate themselves according to their agreement level in 12 statements. These statements were taken from electoral platforms presented by parties during the electoral campaign and could be clearly associated with political positions along the left—right axis.

The cross ideological nature of Argentine electoral politics [18, 19] does not allow us to base the questionnaire on the left-right axis [20]. This does not imply that the 2015 presidential election lacked polarization. Argentina is a relatively young democracy that has held eight presidential elections since 1983. A two-party system has historically dominated politics, namely the *Peronismo* (Peronist Party also known as *Partido Justicialista* or PJ) and the *Unión Civica Radical* (UCR). Both parties hold factions with distinct right and left ideologies within them. After three consecutive terms of peronist government (i.e., Nestor Kirchner from 2003 to 2008 and Cristina Fernandez de Kirchner from 2008 to 2016) a strong division developed between those that supported and those that were very critical of this period [21]. Public discourse and media were highly polarized. There was a clear political fracture framing the electoral race [22], which allowed us to replicate the Swedish experiment in dichotomic terms: incumbent party vs. opposition (the opposition being an electoral coalition between a local party from the Capital in alliance with the declining UCR).

A further difference with the Swedish case is that we had no access to party platforms with a detailed government’s plan, even after the winner of the first round (incumbent candidate Daniel Scioli) took more extreme positions to present himself as the antithesis of his opponent Mauricio Macri. In order to cope with this problem, we selected statements for the questionnaire according to the support or opposition they expressed to the then president Cristina Fernandez Kirchner, which addressed directly the key issues that lead to the political fracture in Argentinian voting inclinations [23]. This allowed us to base the statements on unambiguous policy positions instead of the fluctuating political discourse of the campaign.

In sum, there were both factual and theoretical reasons to use a different political breakout for the selection of the statements and the organization of the questionnaire used in the experiments. In Argentina, the long-term organization of electoral politics, which does not clearly fit in the left right division, resulted in a different breakout: incumbents and challengers.

## 3. Methods

### 3.1 Questionnaire

In both experiments, participants were asked at the start of the questionnaire how politically engaged they were (on a four-options discrete scale from “very engaged” to “not engaged at all”), for whom they intended to vote in the second round (Scioli, Macri, blank, or contested), and their reported ideology in a continuous scale with the two ends being “Scioli” and “Macri”. Age, gender, and education were also asked.

As in the Swedish experiment, the main survey consisted of 12 salient political issues where the two sides held opposing views. We asked the participants to indicate their level of agreement with the statements on a continuous scale where the midpoint represents ‘middle ground’, the left endpoint means ‘absolutely disagree’, and the right endpoint means ‘absolutely agree’. To quantify the level of agreement with a statement, we calculated the distance of the indicated point to the midpoint and rescaled it to a 100-points scale, where 0 is neither agree nor disagree and 100 is either absolutely agree or disagree. The full questionnaire is shown and translated to English in supplementary material S1 File.

### 3.2 Experiment 1

In this experiment, participants were asked to fill a questionnaire concerning their political positions and vote intention. The study was carried out from November 15^th^ to 22^th^, 2015. Ages ranged from 18 to 81 (M: 42, SD: 18). Participants were recruited in several different locations in the city of Buenos Aires (Argentina’s Federal District).

Unknown to participants, experimenters used a magic ‘trick’ to switch the questionnaire after all answers were given. The new answers, produced by the experimenter while the participant was answering the questionnaire, were in some cases changed to their opposite. Participants were then asked to justify some of the manipulated and also some of the non-manipulated replies. Then the experimenter used a semi-transparent template to sum up which side of the political spectrum the participant favored, placing her in the opposite side relative to her voting intention. Finally, participants were asked to report again on their voting intentions and were debriefed about the real nature of the experiment.

### 3.3 Experiment 2

We designed a web based version of the survey, supported in both desktop and mobile devices, with the purpose of greatly increasing the number of participants in the study. Our web based strategy also meant conducting a massive experiment without the need of an interviewer, resulting in a highly scalable, cost effective, data gathering tool.

The survey was posted in social media (Twitter and Facebook), during three days, by “El Gato y La Caja” (elgatoylacaja.com), an online community for the public communication of science. Participants were randomly assigned to the treatment or control group. In the control group, none of the participants’ choices were manipulated.

Participants answered the same 12 questions as in Experiment 1, and afterwards we randomly selected four of their answers, presented them one by one, and asked them if they wanted to change their responses. In each screen, we rephrased the question and showed the participant’s answer (i.e., the indicated point in the agreement line), but in the treatment group the second and fourth answers were changed to the opposite side in the agreement scale (in the control group none of the answers were manipulated). We then presented a new question asking on the level of confidence with the answer in a continuous scale from 0 to 100. Then a statement saying “If you were given the chance, would you like to change your answer?”. Independently of the reported agreement level, the manipulation was implemented in the following way: in a scale from 0 to 100 (where 0 is completely disagree and 100 is completely agree), the agreement was changed to 40 if the original was between 50 and 100 and to 60 if it was between 0 and 50. A proxy for conscious detection in manipulated trials was the participant’s intention to change the manipulated answer. It is important to note that confidence is measured after the manipulation (in M trials) and prior to the question giving the chance to change the M answer. It is not confidence in the first response but, rather, on a possible manipulated answer. As a result, low confidence in manipulated answers may imply that participants detect that there is something wrong with the response we are presenting to them.

At the end of the experiment, we informed participants that, after implementing a complex algorithm, we found that their compass score showed that their opinions were closer to the candidate they didn’t intend to vote, regardless of their answers or detection of manipulations. We then enquired again about their voting intentions. We purposely included several buzz words (*multi dimension*, *machine learning*, etc.) in order to generate the illusion of scientific validity, complexity, and accuracy.

As in Experiment 1, participants were asked to indicate their gender, education, age, vote intention, political engagement, and ideological location. They were also asked to indicate their certainty in their voting intention in a continuous scale. All these parameters have shown to affect political and ideological opinions and polarization (see, for instance, [24–26]). Table 1 summarizes all the collected variables used in the analysis.

**Table 1:** Data collected in Experiment 2.

Variable	Description
At the beginning of the survey	
Education	A discrete variable with 5 values, from primary school (0) to graduate (4).
Age	Integer
Gender	Male, female, or other.
Vote Intention	A categorical variable with the names of the two presidential candidates running in the Argentinian 2015 election.
Certainty in Voting Intention	A continuous variable, expressed in a line, on the subjects reported certainty in voting intention previous to the experiment. Rescaled offline from 0 to 100, where 0 is completely uncertain and 100 is completely certain.
Ideological Location	A continuous variable, expressed in a line from Macri to Scioli, in which the subject was asked to place him/herself. We measure the distance of the indicated point to the midpoint and rescale it to a 100-points scale to quantify its strength.
Main Survey	
Agreement level	A continuous variable, expressed in a line, on the agreement level for each of the 12 statements. The left endpoints mean absolutely disagree, the right endpoints means absolutely agree, and the midpoints neither agree nor disagree. To measure the agreement level with a statement, we calculate offline the distance of the indicated point to the midpoint and rescale it to a 100-points scale, where 0 is neither agreement nor disagreement, and 100 is absolutely agree or disagree.
Confidence level	A continuous variable from 0 to 100 on the self-reported confidence level on each of the four randomly chosen answers, 2 NM and 2 M.
Willingness to change agreement (Detection Rate in M trials, and Change of opinion in NM ones).	A discrete variable (Yes or No) indicating the desire for changing the agreement level in four answers (2 M, 2 NM). In M trials, “Yes” means one detection occurred, while in NM cases this answer revels a change of opinion or a distraction.

Following the experiment, participants were debriefed about the nature of the experiment and asked not to “spoil it”. As an extra caution, we blocked the participant’s IP in order to prevent duplication.

In both Experiments, data was analyzed using multiple linear models and p-values were derived from the likelihood ratio tests of the full model with the effect in question compared with the full model without the effect in question.

### 3.4 Ethics statement

All subjects gave written informed consent and were naïve about the aims of the studies. All the experiments described in this paper were review and approved by the ethics committee: “Comité de Ética del Centro de Educación Médica e Investigaciones Clínicas “Norberto Quirno” (CEMIC)” qualified by the Department of Health and Human Services (HHS, USA): IRb00001745—IORG 0001315. Collected data and matlab scripts used to analyze it are available for download at https://figshare.com/articles/CBP_Argentina_rar/4296206.

## 4. Results

### 4.1 Experiment 1

56 participants (31 males, 25 females) participate in the experiment. Ages ranged from 18 to 81 (M: 42, SD: 18). Participants justified on average 3.3 out of 12 responses (SD = 1.6). Across all participants, we obtained 184 justifications of a total of 672 responses (12 for each of the 56 participants). Out of these, 131 were M and 53 NM. Participants detected that there had been a manipulation in 40% (53) of 131 M trials. 48 participants (88%) were deceived by at least one of the manipulations. When participants detected that there was something wrong in a response, they justified the perceived discrepancy as their own mistake. None of them suspected that there was a "trick" involved and/or that their responses had been manipulated.

None of the participants who initially expressed a voting intention for either presidential candidates changed their voting intention at the end of the experiment (i.e., none of them endorsed the compassed score). Anecdotally, debriefing showed that the main reasons why participants did not change their political voting intentions were mainly: 1) strong emotional feeling that prevented them from voting for the other party and 2) roundabout justifications of why our questions did not touch on their political beliefs.

We ran multiple linear models to evaluate whether gender, age, education, voting intention, self-reported political engagement, strength of ideological location, agreement level, and their interactions, had a significant effect on the detection rate in M statements. The only regressor showing significant predictive power was agreement level (slope = 0.0026, SE = 0.0009, t = 2.7, p < 0.007). The mean detection rate for agreement levels varying in the full range from 0 to 100 increases from 0.14 to 0.4. This result is comparable to the findings by Hall et al. in [14]. Also, as found in previous choice blindness studies [8–10, 13, 14, 27], none of the other factors showed a significant effect.

In sum, our results replicated one of the main findings of the Swedish experiment [14]: participants were largely unaware of manipulations in their expressed political opinions. However, we found a striking difference with the Swedish study. Results from our experiment reliably and categorically showed that, although the aggregated responses after the manipulation clearly suggested to participants that they should change their voting intention, none of our participants agreed to do so.

### 4.2 Experiment 2

#### 4.2.1 Participants

**Fig 1:**
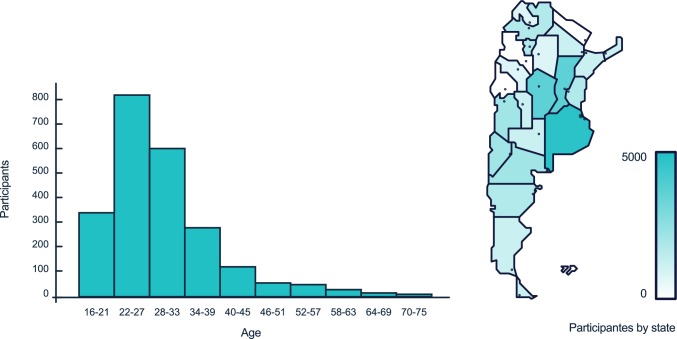
(left) Age distribution and (right) geographical location of the participants.

The aim of the second experiment was to replicate the main findings of Experiment 1, and to look for fine grain effects within the data, which can only be found in a much larger sample. Among the 4537 participants that started the online version of the experiment (a total of 6553 clicked on the link, but 2016 did not start), a total of 3106 finished it. Of the participants included in the analyses, 3054 responded both demographic questions (age and education) and a total of 2325 reported gender information—not a mandatory question—(male: 1099; female: 1226). Geographical and age distributions are shown in Fig 1. Locations were provided by the server that hosted the experiment. Ages ranged from 16 to 75 years (M: 29.4 years; SD: 8.6 years).

**Fig 2:**
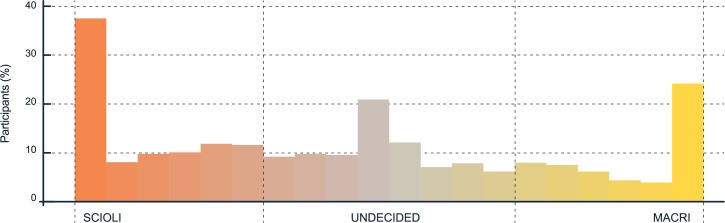
Participants self-reported ideological location.

Participants’ voting intentions were distributed evenly between candidates and were highly polarized with a large number of participants reporting ideological locations in the extremes (Fig 2). In the final result of the presidential election, the opposition candidate won by about a 2.80% margin (51.40% to 48.60%)

#### 4.2.2. Endorsement of compass score

Results were in line with those of Experiment 1. A chi-squared test comparing the amount of change in voting intentions between the treatment group (5% of the sample) and the control group (3.9%) was not significant (p = 0.24). A second analysis between incumbent (5.7%) and opposition (4.7%) voters was also not significant (p = 0.28).

#### 4.2.3. Correction of manipulated answers

Among the participants that completed the experiment, 74% in the treatment group detected the manipulation in at least one of two questions, and 36% did not detect the manipulation in neither of them. In total, collapsing across all 4848 M questions, 55% were not detected; a number comparable with what we obtained in Experiment 1. Overall, this pattern of results confirmed that there was a high level of deception in political statements. Completion rate was higher in the control group than in the treatment group. For the control, 634 participants completed the study (27% attrition rate) and for the treatment group 2472 participants completed the study (33% attrition rate), a significant difference according to chi-squared test (p = 4.3741e-04). The lower rate of participation in the treatment group could be attributed to differences in conscious and unconscious detection; however, considering that the difference in attrition rate between groups is rather small, it has limited impact on the analyses or the interpretation of results reported below.

An advantage of this experiment is that the large sample size allowed us to explore the circumstances (who, when, and why) that lead to higher rates of deception. We measured eight variables (listed in Table 1); these included demographical variables, self-reported ideological variables, and the degree to which participants adhered to each of their stated opinions. We reasoned that, since these variables affect political and ideological opinions and polarization [24–26, 28, 29], they might also affect detection rates. In order to explore this possibility, we performed an exhaustive general lineal model that included all the variables and their pair interactions as potential regressors to account for the variability in the level of deception. Results showed that the model accounts for 47% of the variability in detection rates (R^2 for the full model was .47). Four main regressors (confidence in the manipulated response, agreement level in the response, gender, and voting intention), and the confidence-agreement interaction, had significant predictive power on the detection rate (summarized in S2 File). To identify quantitative estimates of the parameters, we performed a restricted multiple linear regression analysis, including as regressors only the variables and the interaction found to be significant in the first full model. This reduced model, reported in Table 2, verified that, as expected, all of them had significant predictive power (R^2 = .47, p<0.001 in all five cases).

**Table 2:** parameters of the multiple linear regression for detection rates (from 0 to 1), including only the regressors shown to have significant predictive power. Estimate intercept (.58) is the detection rate for 0 confidence, 0 agreement level, females and opposition voters.

	Estimate	SE	tstat	pvalue
Intercept	0.58	0.02	-2.5	0.012
Confidence	-0.0063	0.0003	19.2	3.183e-79
Agreement level	0.0040	0.0003	7.7	2.0351e-14
Voting intention	0.0333	0.011	3.1	0.002
Gender	-0.0368	0.011	3.1	0.0006
conf:agreem	1.97e-05	4.17e-06	4.7	2.42e-06

**Fig 3:**
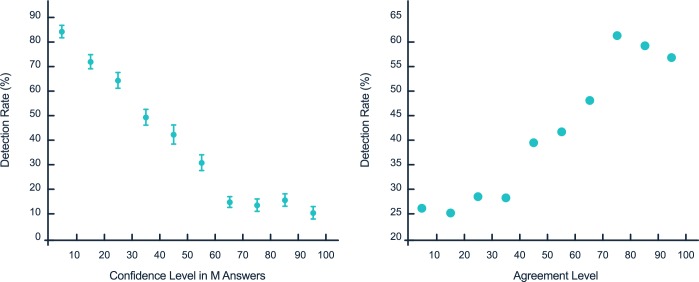
(left) detection rates as a function of the confidence level in M replies. Detection rates change by 80% according the confidence level, using intervals of 10 (R^2 = 0.9). Error bars indicate confidence intervals. (rigth) Detection rates change by more than 40% according to the agreement level. Error bars, indicating confidence intervals, are smaller than the data points size.

To observe the pattern of each of these individual dependencies, we next zoomed into the detection rate data. The strongest effect on detection rate was produced by variations in confidence level in the manipulated responses; detection rate changed from ~85% for low confidence to ~5% for high confidence (Fig 3, left). Note that the confidence level was asked after the manipulation and, as a result, low confidence (which implies that participants doubt and do not trust the manipulated response) is associated with higher detection. This finding indicates that the majority of participants consistently expressed lack of deception with 1) a discrete measure by which they indicated that they wanted to change the response and 2) by a continuous measure, that reflected doubt and low confidence in the manipulated response. However, as we discuss below, these responses are partly decorrelated, with some subjects expressing low confidence in the manipulated report without explicitly asking to change it.

The second parameter with the strongest effect on detection rate was agreement level. A closer look at the functional dependence of detection rate on agreement (Fig 3, right) shows a non-linearity, indicating a regime of low agreement in which participants were highly deceived by manipulations (detected only at 25% rate). Within this regime, the level of deception was largely independent of agreement.

**Fig 4:**
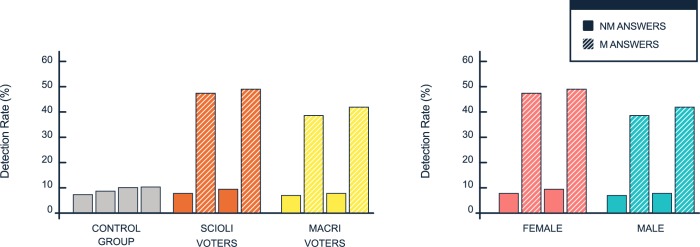
(left) Correction rates for the four questions separated by voting intentions. In the control group, none of the questions were M. In the treatment group, questions two and four were manipulated. (right) Correction rates to the four questions separated by gender.

As the third and fourth parameters with predictive power show, incumbent voters were more difficult to manipulate than opposition voters (48% against 40% detection, p<0.0001, R^2 = 0.00323), and females were more difficult to deceive than men (47% against 43% detection, R^2 = 0.00142, p = 0.01,) (Fig 4).

In the control group (only NM trials) and the NM trials in the treatment group, the rate of corrections was close to 7%. This reflects a small fraction of instances of ‘changes of mind’ without manipulation. This value is consistent in the control group and in the non-manipulated questions of the treatment group and, hence, is likely to reveal a baseline for the degree of ‘change of mind’ without manipulation or a certain degree of noise in the consistency of the responses, given by the uncontrolled conditions of the experiment. This noise, although relatively low, was not observed in Experiment 1 and has not been reported in previous CBP experiments.

Are some opinions easier to deceive?

**Fig 5:**
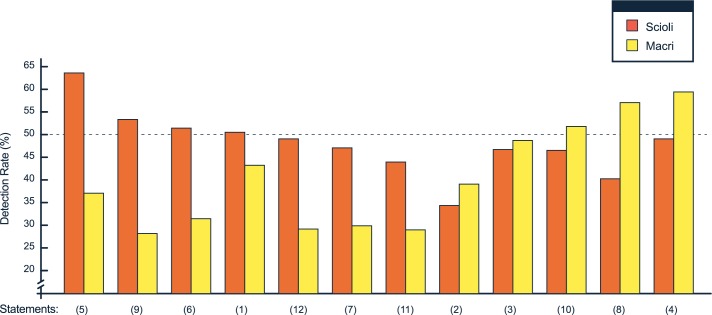
Detection rates for each statement for incumbent and opposition voters. The questions for which we found a significant difference between incumbent and opposition voters (p<0.01 according to a chi-squared test) are 4, 5, 6, 7, 8, 9, 11 and 12. See the translated version of these questions in supplementary material S1 File.

A further aspect of the data we wanted to explore was whether some questions could be harder to manipulate. If that were the case, we expected two possible outcomes: that these questions belonged to the same general kind or that they varied depending on political and ideological location. We reasoned that each voter adheres strongly to a set of political beliefs that provides the backbone for their support of a given party. In consequence, we predicted an interaction between voting intention and deception rate. Indeed, when we analyzed the detection levels separately for each of the 12 claims, we found the expected pattern of differences in deception rate according to voting intentions (See Fig 5).

We then asked to which class of political statements corresponded the six questions in which incumbent voters were less likely to be deceived and, correspondingly, the two questions in which opposition voters were less likely to be deceived. Incumbent voters were most rarely deceived in questions that refer to social plans, distribution of wealth, and other economic measures which were within the core argumentation for these voters (5, 6, 7, 9, 11 and 12; see supplementary material S1 File). Instead, opposition voters were most rarely deceived in questions that relate to accusations of corruption against the government (8 and 4); this issue was at the core of the set of political beliefs of these voters.

#### 4.2.4. Sensitivity of confidence and explicit responses to detect deception

As we described in the previous section, we found overall consistency between confidence judgments and detection rate. We wonder whether confidence judgments could play a role in distinguishing between manipulated and non-manipulated responses; a distinction that cannot be established based on deception measures. In a sense, this analyses evaluates the precision of the confidence system in the same way that studies of metacognition evaluate the capacity of confidence judgments to distinguish between correct and erred responses [30].

Specifically, we were interested in whether, for those participants that did not express deception (i.e. they did not express an explicit intention to change their response), their confidence in M questions will nevertheless be lower than in NM questions. We focused our analyses on the 35% of participants (n = 816) in the treatment group that did not detect any of the two manipulations. We found that 60% of them (n = 492) expressed lower confidence in the two M than in the two NM statements (p < 1e-171 according to a one-tailed binomial test considering the null hypothesis that confidence reports in N and NM trials are equally distributed).

To ensure that these differences could not be explained away by degree in agreement, that we had found to have an effect on confidence, we performed a second analysis. First, we compared confidence levels in undetected M statements against those expressed in M statements, controlling for agreement level. Since in M questions the manipulated agreement was always 40 or 60 (in a scale from 1 to 100, see Section 3.3), we compared the confidence levels in M replies against those expressed in NM replies with agreement between 35 and 45 or between 55 and 65. We found that in undetected manipulations the average confidence level is 74.20 (SE = 0.01, N = 2512), while the in NM replies with comparable agreement is 80.38 (SE = 0.03, N = 758). This significant difference was also observed when the procedure was applied to each of the 12 statement separately; in all cases, the average confidence in NM trials was significantly larger than the average confidence in undetected M trials, even when controlling for agreement level.

## 5. Discussion

The most salient findings of our study are:

1. We observed choice blindness in political decisions in Argentina, giving support to the idea that it is a widespread phenomenon that can be found in different cultures and political systems.
2. We observed the existence of categorical ranges of introspective reports, identified by confidence and agreement levels, separating easy from very hard to manipulate decisions.
3. Contrary to what was observed in Sweden [14], we did not observe changes in voting intentions in response to the manipulation.
4. Many participants did not detect any of the two manipulations, but expressed lower confidence in the M than in the NM statements. This indicates the existence of a new phenomenon; we name this phenomenon unconscious detection of self-deception in direct parallelism to the unconscious metacognition phenomenon in perceptual tasks, and propose it reflects the existence of a neural mechanism unconsciously monitoring our own thoughts.

### 5.1. Change in voting intentions

One important difference between our experiment and the one conducted in Sweden is the low level of endorsement of the compass score in both the live and online versions. In our case, none of the participants agreed to change their voting intentions. This did not happen even for participants showing high levels of deception and, hence, accepting as their own responses that are completely consistent with the voting choice opposite to the one they manifested first. In spite of comparable levels of deception in manipulations of political statements in the Swedish study and ours, deception did not lead to changes in voting intention for Argentinian voters.

### 5.2. Self-deception rates

The detection rates obtained in both experiments were very similar to those obtained in Sweden. In Experiment 2, since we had a large sample size, we could reliably measure dependencies that could not be analyzed in the small sample sizes of previous experiments in the area. We showed that subjects are more likely to detect a manipulated response when they expressed strong agreement level and when they subsequently manifest low confidence in the manipulated response. These findings pose theoretical constrains to the ongoing debate on the scope and limitations of self-confabulation [1, 15]. Our results consistently demonstrate that it is extremely hard, almost impossible, to trick someone when her level of agreement with the statement is very high.

A critical question is why some premises have such a strong level of agreement when others do not. Our study sheds some light on this complicated issue by showing that it is hard to deceive, in political argumentation, a person’s core of foundational premises. However, this study also shows that it is very hard to narrow down the general principles that determine whether a participant will detect a given manipulation or it will go unnoticed. Some insights into how manipulation detection works can be found on studies of perceptual similarity, which show that, when people are asked to compare two stimuli (presented simultaneously), it is easier to classify one stimulus at a time (as is the case in CBP). This perceptual classification finding has been attributed to memory load and to specific aspects of the organization of memory [31]. Memory of a stimulus is often stored with a series of tags; two stimuli that are different in the surface but stored with the same tags (for instance, in the case of face detection could be "a young woman smiling") are susceptible to manipulations that can create complete introspective blindness. Although it was shown that forgetting is not an exhaustive explanation for the CBP [10], our finding that is hard to deceive a person’s core of foundational premises may help to clarify the role of memory and self-confabulation in undetected manipulations.

A second connection to perception findings is that in perception studies violations to categories that are defined by a simple and concise statement (technically their algorithmic or Kolmogorov complexity is minimal) are recognized more easily [32]). It is possible that our finding that statements are clustered into a core might reflect a reduction of complexity and their subsequent encoding into a limited set of categories. In the more complex domain of political, ideological, and moral reasoning, each participant has her own description (language) that can reduce the complexity of a statement to its minimum expression; these are the kind of moral axioms that provide the core of a person’s political beliefs, and are taken as facts and not open to discussion, or in our case, manipulation.

Two others parameters had significant predictive power on detection rate: gender and voting intention. The fact that women were more likely to detect manipulations is consistent with previous findings showing they are less confident, for the same level of performance, in detection of deceptions by others tasks [33]. It is also consistent with a previous finding of our group showing that when watching manipulated pictures of men, women have higher detection rates than men [34].

An additional finding of our study was that incumbent supporters were less likely to be deceived than opposition voters. We speculate that the reason behind this can be attributed to differences in the political attitudes of both groups [18–20]. Incumbent voters belong to the traditional Peronist party, constitute a more cohesive group of voters, have a high degree of political participation, and tend to be informed and up to date with political issues. In contrast, the opposition candidate led a new coalition party that grouped people with heterogeneous political backgrounds and levels of political involvement; they came together mostly around their grievances about the government and not necessarily around opposing political platform. This interpretation is in line with a consistent finding in the literature. Studies on the neurobiological origins of the relation between cognitive biases and the strength of political positions find that supporters with stronger political positions are less likely to be deceived. Arceneaux and collaborators conclude that “individuals are more likely to be persuaded by political arguments that evoke loss aversion via a fearful response” [28]. Chong and Druckman review a number of works and conclude that experiments on political decisions have shown that “Individuals who hold strong attitudes are least susceptible to new information, most likely to counter argue against contrary information, and most likely to recognize information consistent with their prior beliefs” [29, 35].

### 5.3. Unconscious detection of self-deception

We found that 60% of the participants that did not detect any of the two manipulations expressed lower confidence in the two M than in the two NM statements (see 4.2.4.). This finding resembles the unconscious metacognition phenomenon found in perceptual tasks [36–38] (i.e., participants perform above chance in detecting their own errors even on unseen masked trials). Unconscious metacognition occurs when, for instance, participants report they have not seen a target-stimuli (the digit 1, 4, 6 or 9) and perform above chance, not only when comparing the target to the number five (demonstrating subliminal perception), but also when evaluating, through confidence reports, their own performance in the task (demonstrating unconscious metacognition). In our experiment, participants perform above chance when, through confidence reports, they correctly evaluate their performance in detecting manipulations. In light of this parallelism, we name this phenomenon unconscious detection of self-deception. The word “unconscious” only entails here that the participant’s actions indicate lack of awareness of the manipulation.

These findings conflict with the common intuition according to which self-oriented monitoring processes are tightly linked to consciousness. Charles et al. suggest the existence of at least two meta-cognitive systems for performance monitoring in perceptual tasks [38], one of them capable of being deployed non-consciously. The drop in confidence levels in M trials found in our study suggests the existence of a neural mechanism unconsciously monitoring the explanations we give ourselves for the choices we make. These explanations are generated, according to Michael Gazzaniga’s theory, in a neural circuit, in the left side of the brain, he named “the interpreter” [6]. Here we propose the existence of a second and separate mechanism monitoring the interpreter. This proposal is in line with the theory put forward by Daniel Dennett [39] and Stanislas Dehaene [40] that one of the main roles of consciousness—positively selected for during evolution—is to broadcast information (in the form of thoughts) to different brain regions. These regions can then analyze this information and, consciously or unconsciously, identify patterns. In our proposal, the mechanism for the unconscious monitoring of our own thoughts is revealed through the fact that confidence reports drop in undetected M answers, when participants confabulate reasons for the choices they did not make.

## 6. Conclusions

As pointed out by Combs et al [41], politics is often a kind of ‘‘blood sport” in which party affiliation and partisan instincts carry the day more often than bipartisan sentiments. A natural question arises: does learning the principles here studied lead to better decisions and decreases vulnerability towards deception and self-deception?

Recent experiments have shown that learning and reflecting on how we make decisions leads to better decisions [42]. As pointed out by Nisbett in his last book [42], although most researchers are confident that the teaching of statistical principles could have only minimal effects on people´s reasoning in everyday life, this is not the case. Teaching people how to reason statistically with just a few examples in two or three domains is sufficient to improve their reasoning for an indefinitely large numbers of events, even if they bear little resemblance to the ones learned [42].

Moreover, some results show a marked blindness to reasoning when it goes against previously held positions. Political views influence even mathematical performance: when a mathematical operation results in a statistic that goes against a subject´s held political position, he tends to mistake the results [43]. A recent study also shows that defendants of conspiracy theories retain, or even increase, their engagement within the conspiracy echo chamber after interacting with debunking posts [44]. Strong supporters are also likely to dismiss and devalue evidence contrary to their positions and only remember the information that supports their preconceptions. There are two mechanisms that account for this: selective evaluation and selective attribution [45]. The first one is for voters to ignore facts that contradict their evaluation of the incumbent party’s performance when they support it. The second is for voters to adjust their judgments regarding the results of certain policies and who is responsible for the solution of a given problem to their political inclination. The stronger their political inclination, the more acute is the effect of the attribution on their judgments.

In sum, even though there is mixed evidence on the influence of reflection and the study of decision making on our choices, our results open promising new avenues for the study of how reflecting on our choices might affect our political beliefs. A next natural step is to study if teaching the principles here discussed can improve subjects´ understanding of the reasons and factors influencing their choices. As many authors engaged in the free will debate have pointed out [46–48], our decisions are free to the extent that we are informed and aware of the internal and external factors influencing them. In this sense, reflecting on our beliefs may help to develop free societies, which is not just this authors´ desire but a valid endeavor that presents rich grounds for both theoretical and empirical studies in the future.

## Supporting information

S1 File Full questionnaire translated to English. Main survey consisting of 12 salient political issues in Argentina.
(DOCX)

S2 File Experiment 2: parameters obtained through a full model regression. All the variables and their pair interactions shown in Table 1 are considered in the model. P-values were derived from the likelihood ratio tests of the full model with the effect in question compared with the full model without the effect in question.
(DOCX)
