# Supplementary material for: A massive experiment on choice blindness in political decisions: Confidence, confabulation, and unconscious detection of self-deception
Source: PLoS One. 2017 Feb 14;12(2):e0171108. doi: 10.1371/journal.pone.0171108 (PMC5308842; doi:10.1371/journal.pone.0171108)
Supplement: S1 File — Main survey consisting of 12 salient political issues in Argentina. (DOCX) [file pone.0171108.s001.docx]

**Supporting Information S1**

**Full questionnaire translated to English.**

Main survey consisting of 12 salient political issues in Argentina.

1. Debería liberarse por completo el tipo de cambio y eliminarse los controles para la compra y venta de dólares para particulares. (100 macri)

*Currency exchange rates should be completely unregulated as well as limits and controls for the buying and selling of US dollars eliminated.*

2. Argentina debería poner distancia y limitar sus relaciones con el gobierno de Irán. (100 Macri)

*Argentina should set a distance and limit its relation with Iran’s government.*

3. Deberían ampliarse los juicios y alcanzar también a los responsables de los crímenes que cometió la guerrilla. (100 Macri)

*Trials (regarding Argentina’s ‘70 dictatorships) should include crimes commited by local guerrillas.*

4. La pobreza es un fenómeno complejo, es mejor no difundir datos sobre ella hasta que no se encuentre la medida adecuada. (100 Scioli)

*Poverty is a complex phenomenon. No data should be released until a correct way of meassuring is found.*

5. El gobierno debería aplicar la Ley de medios. (100 Scioli)

*The government should apply the ‘Media Law’.*

6. Los subsidios a los servicios públicos deberían mantenerse. (100 Scioli)

*Public service subsidies should be kept.*

7. El acceso a la vivienda debería facilitarse por medio de créditos hipotecarios en lugar del plan procrear. (100 Macri)

*Access to home ownership should be promoted by morgages instead of Procrear.*

8. El INDEC debería volver a ser autónomo. (100 Macri)

*INDEC should go back to being autonomous.*

*9*. Los planes sociales deberían reducirse porque hacen que la gente deje de buscar trabajo. (100 Macri)

*Public service subsidies should be reduced because it leads to people ceasing their job search.*

10. El estado debería seguir usando, a través de la ANSES, los recursos jubilatorios para financiar gasto público. (100 Scioli)

*The State should continue to use retirement funds to finance public spending.*

11. El estado debe obligar a las empresas a que inviertan en la Argentina y por ello sus ganancias deben quedarse en el país. (100 Scioli)

*The State should compel companies to invest in Argentina, therefore, their earnings should be kept within the country.*

12. La Argentina no debería pagarles a los Fondos Buitre. (100 Scioli)

*Argentina should not pay the Vultures.*
