# Supplementary material for: A massive experiment on choice blindness in political decisions: Confidence, confabulation, and unconscious detection of self-deception
Source: PLoS One. 2017 Feb 14;12(2):e0171108. doi: 10.1371/journal.pone.0171108 (PMC5308842; doi:10.1371/journal.pone.0171108)
Supplement: S2 File — All the variables and their pair interactions shown in Table 1 are considered in the model. P-values were derived from the likelihood ratio tests of the full model with the effect in question compared with the full model without the effect in question. (DOCX) [file pone.0171108.s002.docx]

**Supporting Information S2 Table**

Parameters obtained through a full model regression in Experiment 2. All the variables and their pair interactions shown in Table S2 are considered in the model. P-values were derived from the likelihood ratio tests of the full model with the effect in question compared with the full model without the effect in question.

| ***Estimated Coefficients*** | | | | |
| --- | --- | --- | --- | --- |
| ***Estimate*** |  | ***SE*** | ***tStat*** | ***pValue*** |
| ***(Intercept)*** | *0.49195* | *0.099483* | *4.9451* | *7.88E-07* |
| ***Age*** | *0.0054543* | *0.0029961* | *1.8204* | *0.068757* |
| ***Agreement level*** | *0.0044007* | *0.00081247* | *5.4165* | *6.39E-08* |
| ***Education*** | *-0.0054807* | *0.030893* | *-0.17741* | *0.8592* |
| ***Gender*** | *-0.12097* | *0.054845* | *-2.2057* | *0.027456* |
| ***Voting Intentions*** | *0.048138* | *0.05947* | *0.80945* | *0.4183* |
| ***Ideological Location*** | *-0.00091909* | *0.0010299* | *-0.89242* | *0.37221* |
| ***Certainty in Voting Intentions*** | *0.0005751* | *0.00099431* | *0.57839* | *0.56303* |
| ***Confidence Level*** | *-0.0062791* | *0.00071114* | *-8.8296* | *1.47E-18* |
| ***Age:Agreement level*** | *-1.08E-05* | *2.01E-05* | *-0.53995* | *0.58926* |
| ***Age:Education*** | *-0.00069285* | *0.00060498* | *-1.1452* | *0.25217* |
| ***Agreement level:Education*** | *6.88E-05* | *0.00021146* | *0.32522* | *0.74503* |
| ***Age:Gender*** | *-0.00053034* | *0.0012467* | *-0.42539* | *0.67057* |
| ***Agreement level:Gender*** | *0.00057398* | *0.00033106* | *1.7338* | *0.083026* |
| ***Education:Gender*** | *0.0071591* | *0.013687* | *0.52305* | *0.60097* |
| ***Age:Voting Intentions*** | *-0.00088916* | *0.0012689* | *-0.70071* | *0.48352* |
| ***Agreement level:Voting Intentions*** | *0.00036626* | *0.0003441* | *1.0644* | *0.2872* |
| ***Education:Voting Intentions*** | *-0.0095802* | *0.014162* | *-0.67645* | *0.49879* |
| ***Gender:Voting Intentions*** | *0.012968* | *0.022778* | *0.56933* | *0.56916* |
| ***Age:Ideological Location*** | *1.91E-05* | *2.00E-05* | *0.95196* | *0.34117* |
| ***Agreement level:Ideological Location*** | *-5.85E-06* | *5.47E-06* | *-1.0689* | *0.28515* |
| ***Education:Ideological Location*** | *-6.00E-05* | *0.00021998* | *-0.27255* | *0.78521* |
| ***Gender:Ideological Location*** | *-0.00012935* | *0.00036653* | *-0.35291* | *0.72417* |
| ***Voting Intentions:Ideological Location*** | *0.00023584* | *0.00037516* | *0.62864* | *0.52961* |
| ***Age:Certainty in Voting Intentions*** | *-2.29E-05* | *2.61E-05* | *-0.87685* | *0.38061* |
| ***Agreement level:Certainty in Voting Intentions*** | *-6.46E-06* | *6.45E-06* | *-1.0005* | *0.3171* |
| ***Education:Certainty in Voting Intentions*** | *0.0002417* | *0.00027363* | *0.88332* | *0.37711* |
| ***Gender:Certainty in Voting Intentions*** | *0.00071635* | *0.00043216* | *1.6576* | *0.097462* |
| ***Voting Intentions:Certainty in Voting Intentions*** | *-2.62E-05* | *0.00045073* | *-0.058104* | *0.95367* |
| ***Ideological Location:Certainty in Voting Intentions*** | *2.06E-06* | *7.35E-06* | *0.28063* | *0.77901* |
| ***Age:Confidence Level*** | *-1.90E-05* | *1.54E-05* | *-1.2395* | *0.21521* |
| ***Agreement level:Confidence Level*** | *-1.81E-05* | *4.28E-06* | *-4.2224* | *2.46E-05* |
| ***Education:Confidence Level*** | *0.00019236* | *0.00016852* | *1.1414* | *0.25375* |
| ***Gender:Confidence Level*** | *-0.00022958* | *0.00027127* | *-0.84632* | *0.39742* |
| ***Voting Intentions:Confidence Level*** | *-8.35E-05* | *0.00028054* | *-0.29778* | *0.76589* |
| ***Ideological Location:Confidence Level*** | *6.48E-06* | *4.44E-06* | *1.4612* | *0.14403* |
| ***Certainty in Voting Intentions:Confidence Level*** | *-3.00E-06* | *5.46E-06* | *-0.55017* | *0.58223* |
